# The diagnostic performance of shear wave speed (SWS) imaging for thyroid nodules with elasticity modulus and SWS measurement

**DOI:** 10.18632/oncotarget.14534

**Published:** 2017-01-06

**Authors:** Dan Wang, Ya-Ping He, Yi-Feng Zhang, Bo-Ji Liu, Chong-Ke Zhao, Hui-Jun Fu, Qing Wei, Hui-Xiong Xu

**Affiliations:** ^1^ Department of Medical Ultrasound, Shanghai Tenth People's Hospital, Ultrasound Research and Education Institute, Tongji University School of Medicine, Shanghai 200072, China; ^2^ Thyroid Institute, Tongji University School of Medicine, Shanghai 200072, China; ^3^ Shanghai Center for Thyroid Diseases, Shanghai 200072, China; ^4^ Department of Pathology, Shanghai Tenth People's Hospital, Tongji University School of Medicine, Shanghai 200072, China

**Keywords:** shear wave speed imaging, thyroid nodule, elastic modulus, ultrasound, elastography

## Abstract

To evaluate the diagnostic performance of a new technique of shear wave speed (SWS) imaging for the diagnosis of thyroid nodule with elasticity modulus and SWS measurement. 322 thyroid nodules in 322 patients (216 benign nodules, 106 malignant nodules) were included in this study. All the nodules received conventional ultrasound (US) and SWS imaging (Aplio500, Toshiba Medical Systems, Japan) before fine-needle aspiration (FNA) and/or surgery. The values of E-max and E-mean with elastic modulus (61.27 ± 36.31 kPa and 31.89 ± 19.11 kPa) or SWS (4.45 ± 1.49 m/s and 3.26 ± 2.71 m/s) in malignant nodules were significantly higher than those in benign lesions (29.18 ± 18.62 kPa and 15.85 ± 6.96 kPa, or 2.98 ± 0.85 m/s and 2.19 ± 0.42 m/s, all *P* < 0.001). No significant differences in area under the curve (AUC) between the SWS imaging parameters were found (all *P* > 0.05). In multivariate logistic regression analysis, E-max (m/s) with SWS was identified to be the strongest independent predictor for malignant nodules (odds ratio [OR] = 16.760), followed by poorly-defined margin (OR = 7.792), taller-than-wide shape (OR = 3.160), micro-calcification (OR = 2.422), and E-max (kPa) with elastic modulus (OR = 0.914). The AUC was 0.813 for E-max with SWS (m/s) and 0.796 for E-max with elastic modulus (kPa). With cut-off SWS value of 3.52 m/s in E-max, sensitivity of 69.8%, specificity of 81.5%, and accuracy of 77.6% were achieved. SWS imaging is a valuable tool in predicting thyroid malignancy. E-max with SWS measurement is the strongest independent predictor for thyroid malignancy.

## INTRODUCTION

Over the past few decades, the incidence of thyroid carcinoma has considerably increased worldwide [1, 2] whereas the thyroid cancer mortality decreased, which reflects two variations, *i.e*., risk factor exposure and diagnosis/treatment of the disease. The increase in incidence is probably attributed to the advance in detection of this tumor [3], while the decrease of mortality rates in most countries is most likely due to improved diagnosis, management and treatment [4, 5]. Conventional US is a common imaging investigation in diagnosing thyroid malignancy. The US features in indicating malignant thyroid nodules include: solid component, hypoechogenicity, taller than wide shape, irregular margin, no halo, micro-calcifications and intra-nodular vascularity at color Doppler US [6, 7]. However, each characteristic has a varying sensitivity and specificity in predicting thyroid cancer and information about the mechanical properties of benign and malignant nodules is lacking [8, 9].

It is known that tissue stiffness is associated with neoplasia and inflammation, which alters the tissue composition and structure and increases the parenchymal stiffness. Recently, US elastography has emerged as a new tool for evaluating the tissue stiffness. According to the recommendation by World Federation for Ultrasound in Medicine and Biology (WFUMB) guideline for clinical use of elastography on thyroid, elastography techniques can be mainly classified into the following types: (A) Strain imaging, including strain elastography (SE) that evaluates strain in response to external compression (by hand or using cardiovascular pulsation or respiratory motion) and acoustic radiation force impulse (ARFI) imaging wherein the tissue displacement is caused by ARFI excitation from the transducer; (B) Shear wave imaging, including point shear-wave speed (SWS) measurement, SWS imaging, and transient elastography. Their principle are originated from mechanical vibration or pressure or by applying acoustic pressure. [10–12]. US elastography is a useful supplemental tool to conventional US in thyroid evaluation, as evidenced by many literatures and guidelines [10, 13]. However, strain imaging such as SE is limited because of lacking quantitative information and high operator-dependence [14]. SWS imaging appears to have the advantage of operator-independence and real-time operator feedback [15, 16]. A recent meta-analysis identified that of the 131 studies, in which shear wave imaging, point SWS measurement or SWS imaging, was used to evaluate 1867 thyroid nodules in 1525 patients, the pooled sensitivity, specificity, and AUC of shear wave imaging for predicting malignancy were 84.3% (95% confidence interval [CI], 76.9–89.7%), 88.4% (95% CI, 84.0–91.7%), and 93% (95% CI, 90–95%), respectively [17].

Recently, a new SWS imaging was developed (i.e. Toshiba SWS imaging; Aplio 500, Toshiba Medical Systems, Otawara, Tochigi, Japan), which allows the propagation velocities of shear waves to be quantified and mapped. The main feature of this new technique of SWS imaging is that images can be viewed using three different display modes: Speed (shear wave speed, SWS) mode (range, 0–8 m/s); Elasticity (elastic modulus) mode (range, 0–180 kPa) and Propagation (arrival time contour) mode. Unfortunately, up to now, there has been no any review or report on SWS imaging in comparing SWS (m/s) with elasticity modulus (kPa) for diagnosis of thyroid nodules using this technique. As evidenced by recent clinical practice, SWS imaging techniques from different manufacturers might have different cut-off values of the quantitative parameters for predicting malignancy [11, 18]. Therefore, this study was aimed to provide more information for clinicians to select the appropriate SWS parameter for the identification of malignant thyroid nodules.

## RESULTS

### Demographics

The 322 subjects included 79 men (mean age, 50.5 ± 12.6 years; age range, 20.0–75.0 years) and 243 women (mean age, 51.3 ± 12.9 years; age range, 22.0–78.0 years). Age was associated with malignancy (*P* < 0.05) in our cohort, especially in middle-aged people, but sex was not. In general, age in benign group (52.8 ± 12.3y; 20.0–78.0y) was older than that in malignant group (47.9 ± 13.2y; 22.0–78.0y) (*P* < 0.001). In patients with age from 20.0 to 35.0 years and those from 56.0 to 78.0 years, the two subgroups did not show significant differences. However, for those with middle age (36.0–55.0y), the age in benign group (48.3 ± 5.2y; 36.0–55.0y) was older than that in malignant group (45.6 ± 6.7y; 36.0–54.0y) (*P* = 0.017). 169 nodules were located in the right lobe, 147 nodules in the left and 6 nodules in the isthmus. The maximum diameter of malignant nodules (13.1 ± 7.0 mm; range, 6.2–40.0 mm) was not significantly different with that of benign nodules (14.0 ± 9.5 mm; range, 5.0–56.0 mm) (*P* > 0.05). (Table 1)

**Table 1:** Basic demographic characteristics of patients and nodules

	Benign	Malignant	*P* value
**Patient (*n* = 322)**			
**Men/Women**	49/167	30/76	0.274
**Age#**	52.8 ± 12.3 (20.0–78.0)	47.9 ± 13.2 (22.0–78.0)	0.001
**20–35 (*n* = 28/24)**	31.0 ± 4.1 (20.0–35.0)	31.4 ± 3.4 (22.0–35.0)	0.748
**36–55 (*n* = 94/50)**	48.3 ± 5.2 (36.0–55.0)	45.6 ± 6.7 (36.0–54.0)	0.017
**56–78 (*n* = 94/32)**	63.7 ± 5.7 (56.0–78.0)	63.8 ± 5.6 (57.0–78.0)	0.967
**Nodule (*n* = 322)**	216	106	
**Location**			0.668
**Left**	99	48	
**Right**	114	55	
**Isthmus**	3	3	
**Nodule size (mm)#**	14.0 ± 9.5 (5.0–56.0)	13.1 ± 7.0 (6.0–40.0)	0.332
**≤ 10 (*n* = 84/39)**	6.4 ± 1.3 (5.0–10.0)	7.5 ± 0.9 (6.0–10.0)	0.000
**11–20 (*n* = 82/50)**	12.9 ± 2.7 (11.0–20.0)	13.1 ± 2.6 (11.0–20.0)	0.822
**> 20 (*n* = 50/17)**	28.7 ± 7.6 (21.0–56.0)	26.2 ± 6.4 (21.0–40.0)	0.229

**Note.**—Data are means ± standard deviations.

### FNA and surgery

**Figure 1:**
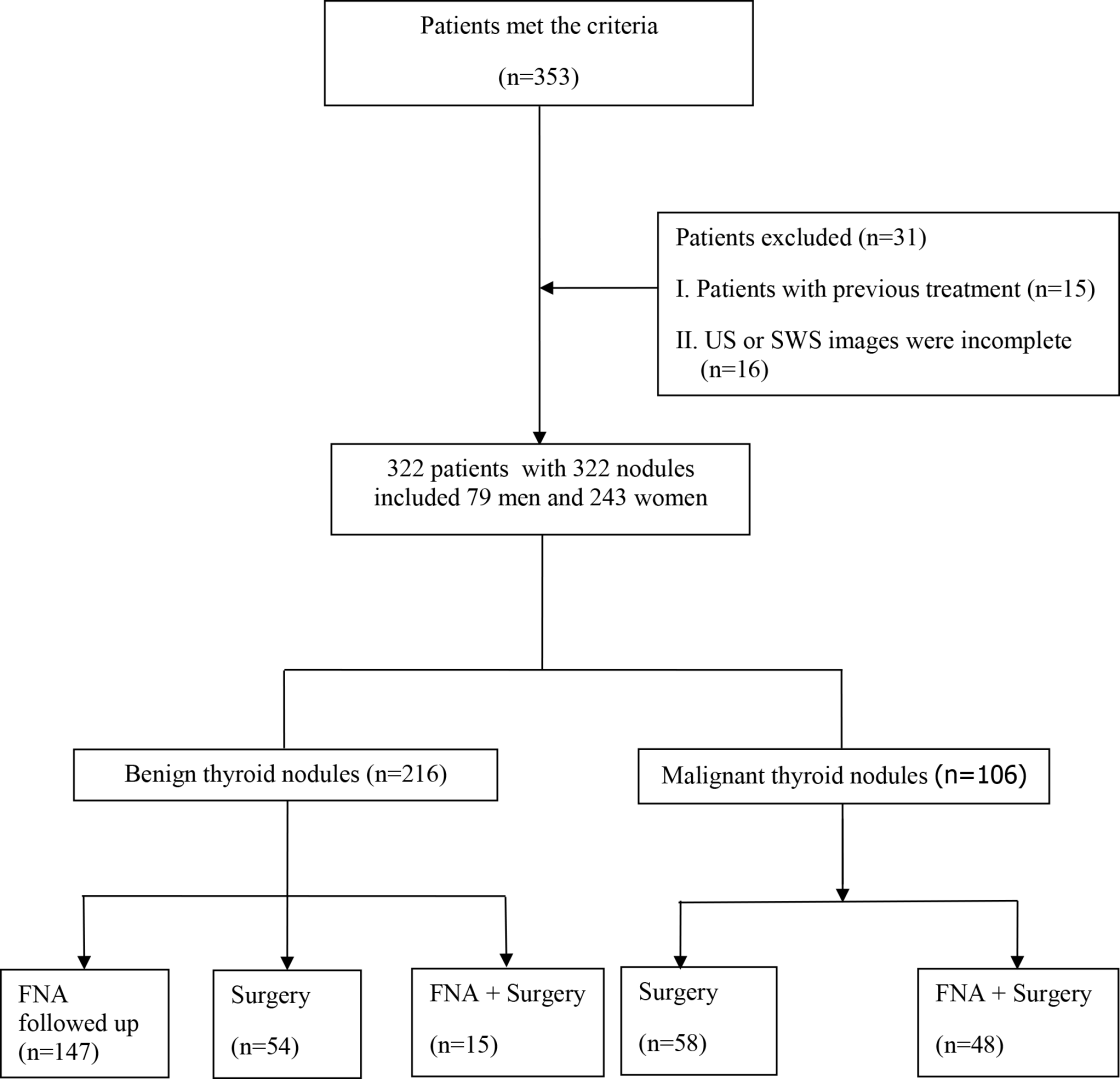
Flowchart for the selection of thyroid nodules

Among the 322 thyroid nodules (TNs), 106 were malignant and 216 were benign. Of them, 175 nodules were confirmed by pathological results and the remaining 147 nodules were confirmed by FNA and follow-up. For the nodules with benign FNA cytological results, they were confirmed by US follow-up and no change was observed on US during a follow-up period of more than 6 months (Figure 1). Of the 175 nodules with pathological results, 53 were nodular goiters, 1 was adenoma and 15 were Hashimoto nodules; for malignant lesions, 105 nodules were diagnosed with papillary thyroid carcinomas and the remaining one nodule was diagnosed with medullary thyroid carcinoma.

### Conventional US

The diagnostic performances of the main features of conventional US in predicting malignancy are shown in Table 2, which include solid component, hypoechogenicity, poorly-defined margin, taller than wide shape, halo sign, micro-calcification, and vascularity on color Doppler US. Poorly-defined margin (82.1% sensitivity and 69.4% specificity, *P* < 0.001) was the most predictive US feature for malignancy. In addition, a high sensitivity was found with solid component (89.6%) whereas its specificity was low (34.3%). Conversely, a high specificity was found with taller-than-wide shape (81.0%) whereas its sensitivity was only 43.4 %. Halo sign and vascularity on color Doppler US were not associated with malignancy (*P* > 0.05) in our cohort and showed low diagnostic performance in Table 2.

**Table 2:** Conventional US and SWS imagine features in predicting thyroid malignancy

Parameter	Benign Nodules *n* = 216 (%)	Malignant Nodules *n* = 106 (%)	*P* value	Cut-off value	SEN (%)	SPE (%)	PPV (%)	NPV (%)	ACU (%)	AUC^&^
Solid Component		< 0.001		89.6	34.3	40.1	87.1	52.5	0.619 (0.564–0.673)
Yes	142 (65.7)	95 (89.6)								
No	74 (34.3)	11 (10.4)								
Hypoechogenicity			< 0.001		70.8	52.3	42.1	78.5	58.4	0.615 (0.560–0.669)
Yes	103 (47.7)	75 (70.7)								
No	113 (52.3)	31 (29.3)								
Taller-than-Wide Shape			< 0.001		43.4	81.0	52.9	74.5	68.6	0.622 (0.567–0.675)
Yes	41 (19.0)	46 (43.4)								
No	175 (81.0)	60 (56.6)								
Microcalcification			< 0.001		62.3	75.0	55.0	80.2	70.8	0.686 (0.633–0.737)
Yes	54 (25.0)	66 (62.3)								
No	162 (75.0)	40 (37.7)								
Poorly-defined Margin			< 0.001		82.1	69.4	56.9	88.8	73.6	0.758 (0.707–0.803)
Yes	66 (30.6)	87 (82.1)								
No	150 (69.4)	19 (17.9)								
Halo sign			0.092		17.0	74.5	24.7	64.7	55.6	0.542 (0.486–0.598)
Yes	55 (25.5)	18 (17.0)								
No	161 (74.5)	88 (83.0)								
Vascularity			0.448		34.9	69.4	35.9	68.5	58.1	0.522 (0.466–0.577)
I–II	150 (69.4)	69 (65.1)								
III–IV	66 (30.6)	37 (34.9)								
SWS imagine(kpa)										
E-max	29.18 ± 18.62	61.27 ± 36.31	< 0.001	47.00	59.4	89.8	74.1	81.9	79.8	0.796 (0.748–0.839)
E-mean	15.85 ± 6.96	31.89 ± 19.11	< 0.001	23.00	60.4	91.2	77.1	82.4	81.1	0.807 (0.760–0.849)
SWS imagine(m/s)										
E-max	2.98 ± 0.85	4.45 ± 1.49	< 0.001	3.52	69.8	81.5	64.9	84.6	77.6	0.813 (0.766–0.854) (0.748–0.839)
E-mean	2.19 ± 0.42	3.26 ± 2.71	< 0.001	2.46	67.9	83.3	66.7	84.1	78.3	0.800 (0.748–0.839)

Note–numbers in parentheses are percentages

Data are means ± standard deviations

SWS imagine = shear wave speed imagine; E-max = the max value of shear wave speed imagine; E-mean = the mean value of shear wave speed imagine ;

N/A = not apply

& Note–numbers in parentheses are 95% confidence interval (CI)

AUC = area under receiver operating characteristic curve; ACU = accuracy; NPV = negative predictive value; PPV = positive predictive value; SEN = sensitivity; SPE = specificity

### SWS imaging

**Figure 2:**
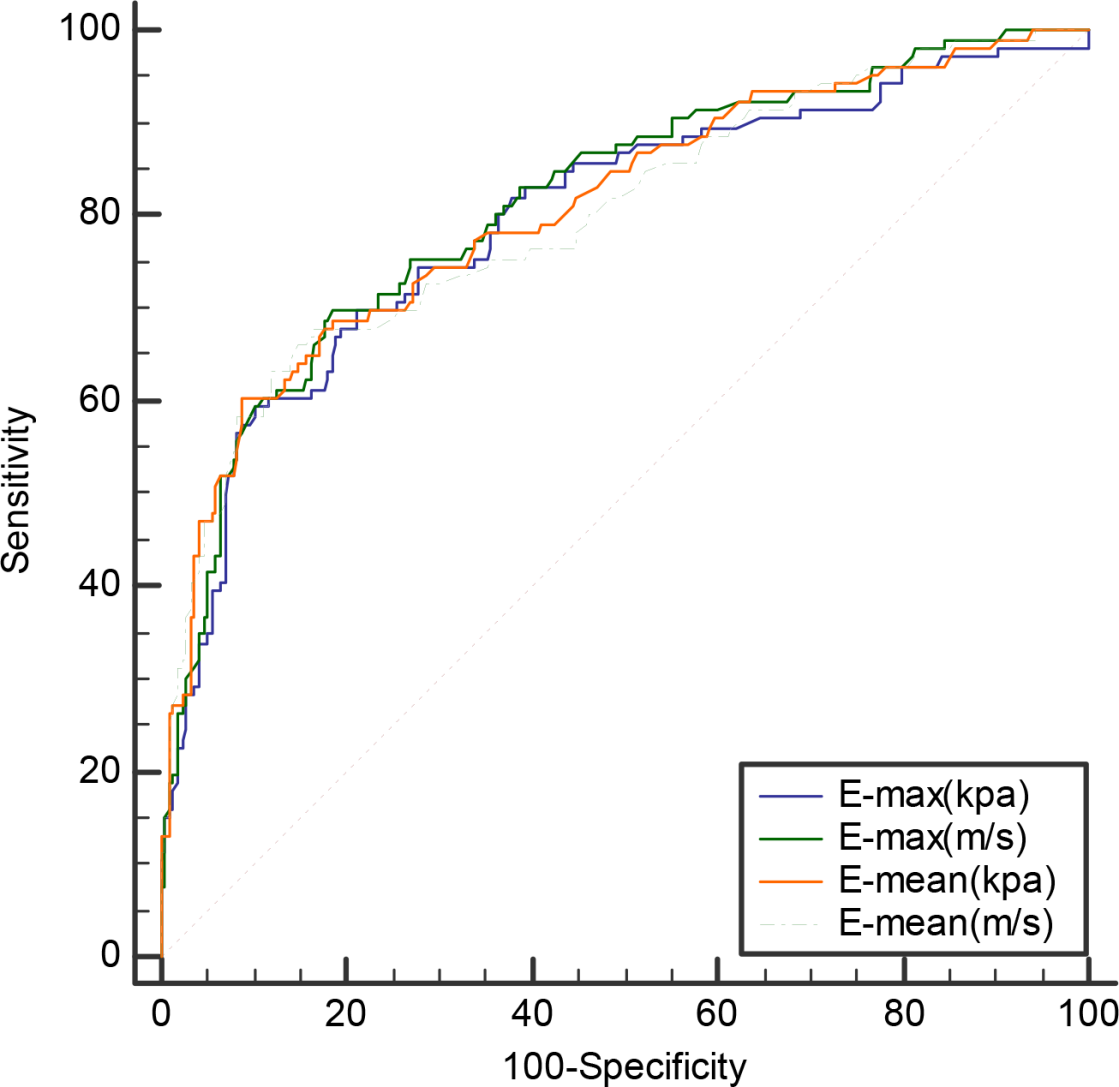
Receiver operating characteristic (ROC) curves for differentiating benign and malignant nodules with SWS imagine

The E-max and E-mean values of SWS imaging with elasticity modulus (61.27 ± 36.31 kPa and 31.89 ± 19.11 kPa) or SWS (4.45 ± 1.49 m/s and 3.26 ± 2.71 m/s) in malignant nodules were significantly higher than those in benign lesions (29.18 ± 18.62 kPa and 15.85 ± 6.96 kPa; or 2.98 ± 0.85 m/s and 2.19 ± 0.42 m/s, all *P* < 0.001) (Table 2). The AUC of E-max and E-mean were 0.796 (95% CI: 0.748–0.839) and 0.807 (95% CI: 0.760–0.849) with elasticity modulus, 0.813 (95% CI: 0.766–0.854) and 0.800 (95% CI: 0.748–0.839) with SWS, respectively. (Table 2) No significant differences of AUC between the SWS imaging parameters were found (all *P* > 0.05) (Figure 2). Diagnostic performance in terms of the corresponding sensitivity, specificity, accuracy, positive predictive value (PPV) and negative predictive value (NPV) for optimal diagnostic cut-off values are listed in Table 4. Among these SWS imaging parameters, E-max with SWS (m/s) showed relatively higher AUC with an optimal cut-off SWS value of 3.52 m/s, achieving 69.8 % sensitivity, 81.5% specificity, 77.6% accuracy, 64.9% PPV and 84.6% NPV. (Table 2).

**Table 3:** The diagnostic performances of SWS imagine in predicting thyroid malignancy with different sizes

SWS indices	Cut-off value		SEN (%)	SPE (%)	PPV (%)	NPV (%)	ACU (%)	AUC^&^	P*	P^#^	P^&^	P^^^
**Nodule size ≤ 10 mm (*n* = 123)**									**0.528**	**0.083**	**0.031**	**0.003**
**E-max**	kpa	24.4	76.9	76.2	60.0	87.7	76.4	0.776 (0.692,0.846)				
	m/s	2.82	76.9	72.6	56.6	87.1	74.0	0.788 (0.705,0.857)				
**E-mean**	kpa	19.3	43.6	88.1	63.0	77.1	74.0	0.696 (0.607,0.776)				
	m/s	2.61	41.0	89.3	64.0	76.5	74.0	0.672 (0.581,0.754)				
**11 ≤ Nodule size ≤ 20 mm (*n* = 132)**									**0.668**	**0.334**	**0.583**	**0.435**
**E-max**	kpa	46.9	80.0	91.5	85.1	88.2	87.1	0.894 (0.829,0.941)				
	m/s	3.92	78.0	92.7	86.7	87.4	87.1	0.895 (0.830,0.942)				
**E-mean**	kpa	22.5	74.0	91.5	84.1	85.2	84.8	0.883 (0.815,0.932)				
	m/s	2.45	82.0	84.1	76.0	88.5	83.3	0.876 (0.808,0.927)				
**Nodule size >20 mm (*n* = 67)**									**0.193**	**0.685**	**0.251**	**0.911**
**E-max**	kpa	52.4	88.2	82.0	62.5	95.3	83.6	0.810 (0.696,0.896)				
	m/s	4.13	94.1	84.0	66.7	97.7	86.6	0.883 (0.781,0.949)				
**E-mean**	kpa	27.8	70.6	92.0	75.0	90.2	86.6	0.886 (0.785,0.951)				
	m/s	2.67	76.5	88.0	68.4	91.7	85.1	0.877 (0.774,0.945)				

AUC=area under receiver curve; ACU,=accuracy; NPV=negative predictive value; PPV=positive predictive value; SEN=sensitivity; SPE= specificity; SWS=shear wave speed; E-max = the max value of shear wave speed imagine; E-mean = the mean value of shear wave speed imagine ;

^&^ Note–numbers in parentheses are 95% confidence intervals (CI)

P* AUC in comparison with that of E-max between elasticity and speed

P^#^ AUC in comparison with that of E-mean between elasticity and speed

P^&^ AUC in comparison with that of E-max elasticity and E-mean elasticity

P^^^ AUC in comparison with that of E-max speed and E-mean speed

**Table 4:** Logistic regression analysis of suspicious conventional US features and SWS imagine indices for predicting malignancy

Parameter	β	SE	OR (95% CI)	*P* Value
**Univariate analysis**				
Conventional US risk factor				
Poorly-defined Margin	2.342	0.293	10.407 (5.8585,18.486)	0.000*
Microcalcification	1.599	0.255	4.950 (3.005, 8.154)	0.000*
Solid Component	1.504	0.349	4.501 (2.270,8.924)	0.000*
Taller-than-wide shape	1.186	0.262	3.272 (1.959,5.466)	0.000*
Hypoechogenicity	0.976	0.253	2.654 (1.616,4.360)	0.000*
SWS imagine risk factor				
E-mean ≥ 2.46 m/s	2.108	0.283	8.229 (4.727,14.327)	0.000*
E-max ≥ 3.52 m/s	1.074	0.137	2.296 (2.238,3.825)	0.000*
E-mean ≥ 23.00 kpa	0.117	0.017	1.124 (1.088,1.161)	0.000*
E-max ≥ 47.00 kpa	0.043	0.006	1.044 (1.032,1.056)	0.000*
**Multivariate analysis**				
Conventional US risk factor				
Poorly-defined Margin	2.053	0.439	7.792 (3.295,18.424)	0.000*
Taller-than-wide shape	1.151	0.392	3.160 (1.466,6.812)	0.003*
Microcalcification	0.885	0.367	2.422 (1.179, 4.975)	0.016*
SWS imagine risk factor				
E-max ≥ 3.52 m/s	2.819	1.010	16.760 (2.317,121.257)	0.005*
E-max ≥ 47.00 kpa	0.090	0.046	0.914 (0.835,0.999)	0.045*

SWS imagine = shear wave speed imagine ; E-max = the max value of shear wave speed imagine; E-mean = the mean value of shear wave speed imagine

Note.- ^β^, regression coefficient; SE, standard error; OR, odds ratio; CI, confidence interval

### Subgroup analysis with different sizes

For E-mean with elastic modulus or SWS, the AUC of SWS imaging in nodules ≤ 10 mm was significantly lower than that in nodules 11–20 mm and those > 20 mm (all *P* < 0.05). Furthermore, for E-max with elasticity modulus or SWS, the AUC in nodules ≤ 10 mm was significantly lower than that in 11–20 mm. The comparison of AUCs among the subgroups according SWS imaging parameters as follows: for E-max in kPa, nodules sized ≤ 10 mm vs. 11–20 mm (0.776 vs. 0.894, *P* = 0.040); sized ≤ 10 mm vs. sized > 20 mm (0.776 vs. 0.810, *P* = 0.693); sized 11–20 mm vs. sized > 20 mm (0.894 vs.0.810, *P* = 0.283); for E-max in m/s, (0.778 vs. 0.895, *P* = 0.031); (0.778 vs. 0.883, *P* = 0.120); (0.895 vs. 0.883, *P* = 0.841); for E-mean in kPa, (0.696 vs. 0.883, *P* = 0.002); (0.696 vs. 0.886, *P* = 0.005); (0.883 vs. 0.886, *P* = 0.955); for E-mean in m/s, (0.672 vs. 0.876, *P* = 0.001); (0.672 vs. 0.877, *P* = 0.005); (0.876 vs. 0.877, *P* = 0.987). Based on the optimal cut-off values with different parameters, the sensitivity, specificity, accuracy, PPV and NPV are presented in Table 3.

### Logistic regression analysis for predicting malignancy

In univariate logistic regression analysis, the main suspicious conventional US finding and the cut-off value of SWS imaging were statistically significant predictors for malignancy: poorly-defined margin (OR = 10.407, 95% CI: 5.858–18.486, *P* < 0.001), microcalcification (OR = 4.950, 95% CI: 3.005–8.154, *P* < 0.001), solid component (OR = 4.501, 95% CI: 2.270–8.924, *P* < 0.001), taller than wide shape (OR = 3.272, 95% CI: 1.959–5.466, *P* < 0.001), hypoechogenicity (OR = 2.654, 95% CI: 1.616–4.360, *P* < 0.001), and E-mean (m/s) (OR = 8.229, 95% CI: 4.727–14.327, *P* < 0.001), E-max (m/s) (OR = 2.926, 95% CI: 2.238–3.825, *P* < 0.001), E-mean (kPa) (OR = 1.124, 95% CI: 1.088–1.161, *P* < 0.001), E-max (kPa) (OR = 1.044, 95% CI:1.032–1.056, *P* < 0.001) (Table 4).

However, E-max (m/s) with SWS was identified to be the strongest independent predictor for malignant nodules (OR = 16.760, 95% CI: 2.317–121.257, *P* = 0.005), followed by poorly-defined margin (OR = 7.792, 95% CI: 3.295–18.424, *P* < 0.001), taller-than-wide shape (OR = 3.160, 95% CI: 1.466–6.812, *P* = 0.003), micro-calcification (OR = 2.422, 95% CI: 1.179–4.975, *P* = 0.016), and E-max (kPa) with elastic modulus (OR = 0.914, 95% CI: 0.835–0.999, *P* = 0.045). (Table 4).

### Inter-operator and intra-operator consistency of quantitative in E-mean

E-mean expressed in kPa and m/s were used to assess the inter-operator and intra-operator consistency of SWS imagine. The k value of E-mean with kPa was 0.845 (95% CI: 0.801–0.883) for inter-operator consistency while 0.866 (95% CI: 0.824–0.901) for intra-operator consistency. The k value of E-mean with m/s was 0.820 (95% CI: 0.771–0.862) for inter-operator consistency while 0.801 (95% CI: 0.751–0.845) for intra-operator consistency. The k values were all above 0.80, which indicates excellence of intra-operator consistency.

## DISCUSSION

Conventional US has been widely used to predict the risk of malignancy in thyroid nodules and make a decision on whether FNA or surgery is recommended rather than palpation. The probability of malignancy increases as the number of suspicious US features increases. Recently, the ATA guideline has defined high suspicious US features of thyroid nodules including solid hypoechoic nodule or solid hypoechoic component of a partially cystic nodule with one or more of the following features: irregular margins (infiltrative, micro-lobulated), micro-calcifications, taller than wide shape, and Nachiappan et al. had proposed other suspicious features including marked hypo-echogenicity, absence of a hypo-echoic halo and solid composition, extension beyond the thyroid capsule and cervical lymph node metastases [19, 20]. However, the sensitivities and specificities were varying for any single feature [21].

Recently, WFUMB guideline has been released, which demonstrates that elastography is recommended as an additional tool to conventional US and SWS imaging (point SWS and SWS) may be useful in selecting patients with thyroid nodules for surgery and guiding follow-up of lesions with previous results of FNA cytology diagnosed as benign [22]. Up to now, some clinic reviews have reported that SWS imaging is useful for the differential diagnosis of thyroid lesions. E-mean and E-max have been reported to be the most significant parameters, sensitivities ranging from 47.1% to 96.8% and specificities ranging from 71% to 100% [17, 18].

In the present study, we analyzed the SWS parameters in the whole lesion (E-mean) and in the stiffest area of target lesion with a 2-mm-diameter ROI (E-max) and found statistically significant differences between benign and malignant lesions. All SWS imaging parameters of E-mean and E-max, including elastic modulus and SWS were higher for malignant lesions than for benign lesions. We found that E-max (m/s) was identified to be the strongest independent predictor for malignant nodules (OR = 16.760), followed by poorly-defined margin (OR = 7.792), taller-than-wide shape (OR = 3.160), micro-calcification (OR = 2.422), and E-max (kPa) with elastic modulus (OR = 0.914). In concordance with our results, Katarzyna et al. also revealed that E-max was the only SWS imaging independent parameter in differentiation between malignant and benign tumors (OR = 2.95) and for conventional US were irregular margins (OR = 10.82), micro-calcifications (OR = 4.3) [23]. Additionally, their study reported that hypo-echogenicity (OR = 3.13) was related to malignancy. These results indicate that SWS imaging is a valuable tool in predicting thyroid malignancy and E-max with SWS measurement is the strongest independent predictor for thyroid malignancy. For the study of Katarzyna et al., their attention was only focused on the Young's elastic modulus of SWS imaging, not exploring the SWS value. In the present study, we found that E-max (m/s) with SWS ( OR = 16.760) was far better than E-max (kPa) with elastic modulus (OR = 0.914) for predicting malignancy. It is well known that the relationship between the measured SWS (m/s) and elasticity modulus (kPa) is as following : E = 3·ρ·V^2^, wherein E is elastic modulus (kPa), ρ is density, and V is the shear wave propagation speed (m/s). However, the shear wave propagation elastic modulus (kPa) is strongly influenced by the SWS and is also affected by the viscosity (Pa·S). The viscosity affects the physical properties of the tissues, especially in inflammatory diseases, and is considered to affect the physical properties of tissues in many tumorous conditions as well. Therefore, the value of elastic modulus (kPa) is relative, but SWS (m/s) is absolute. SWS (m/s) is more reliable than elastic modulus (kPa).

Up to now, several studies have reported SWS imaging with different parameters for predicting thyroid malignancy. Firstly, according to the Young's elasticity mode with SWS imaging technique (i.e. SuperSonic Imagine, SSI, Aix en Provence, France), Park et al. presented the findings for 476 nodules, including 379 malignant ones, reporting E-max with a cutoff value of 94 kPa and 85kPa of E-mean, with a low sensitivity of approximately 50% and a specificity of approximately 85% [24]. Additionally, Li et al. published different results. In their study, the optimal cut-off values of E-max and E-mean with Young's elastic modulus for predicting malignancy were 53.2 kPa and 34.5kPa, respectively. The sensitivity, specificity, PPV, NPV was 82.1%, 62.3%, 52.1%, 86.4% for E-max, while 83.7%, 77.4%, 63.3%, 89.7% for E-mean [25]. However, in our study, the cut off values were lower than above studies. The optimal cutoff values for predicting malignancy were 23.0 kPa for E-mean, 47.0 kPa for E-max, with a sensitivity of approximately 60% anda specificity approximately 90%, PPV approximately 75%, NPV approximately 80%. The differences were largely due to different cohort population and different SWS imaging techniques used.

On the other hand, according to the SWS mode, Azizis et al. reported the results of SWS imaging with a different technique (i.e. Virtual Touch Tissue Imaging and Quantification, VTIQ; Siemens, Mountain View, CA, USA). In their prospective study evaluates 707 nodules, including 82 malignant nodules. The ROC analysis identified a single cut-off value of 3.54 m/s as the maximum SWS for predicting thyroid cancer. The sensitivity, specificity, PPV, NPV were 79.27% ,71.52%, 26.75%, 96.34%, respectively [26]. In our study, with a cutoff value of 3.52 m/s for E-max, we achieved sensitivity, specificity, PPV, NPV of 69.8 %, 81.5%, 64.9%, 84.6%, respectively. The results of Azizis et al. were similar to our results, which indicated from another aspect that SWS (m/s) might obtain more consistent results among different SWS imaging techniques, in comparison with elastic modulus (kPa).

As far as we know, no studies have been carried out to compare elastic modulus with SWS for SWS imaging, especially in thyroid disease, with no clear reference standards for differentiation to date. Therefore, this aim of this study was to provide evidence for clinicians to select the appropriate SWS parameter under different conditions. In the current study, all the diagnostic sensitivity and specificity of SWS imaging parameters were in the range of 59.4%–69.8% and 81.5%–91.2%, respectively. Another result in the current study should be noticed, the NPV values of four SWS imaging indices were all more than 80% for predicting thyroid malignancy, which is meaningful for clinic work. In other words, if a value of SWS imaging parameter was under the optimal cut-off value, unnecessary FNA might be avoided in approximately 80% thyroid nodules. For the current study, neither of conventional US features demonstrates a balanced sensitivity and specificity in diagnosing thyroid nodules with all AUCs under 0.76 while SWS imaging presents higher diagnostic performance.

Bhatia *et al*. concluded that several factors can influence SWS imaging values, such as lesion size and the position and presence of calcifications [27]. In this study, we specifically explored the influence of nodule size. Although Kim et al. reported that the nodule size did not influence the SWS imaging for either benign or malignant nodules [28]. Bahatia et al. also revealed that the size was not correlated with papillary carcinoma [27]. However, the studies by Liu et al. with real-time SWS imaging and Zhang et al. with point SWS measurement showed relatively inferior diagnostic performance for nodules ≤ 10 mm [18, 29]. Our study was consistent with theirs. For the mean values of SWS imaging, the AUC in nodules ≤ 10 mm was significantly lower than that in nodules > 20 mm, 11–20 mm (all *P* < 0.05). Furthermore, for the max value of SWS imaging, the AUC in nodules ≤ 10 mm was also significantly lower than that in 11–20 mm. It is well known that a malignant nodule equal to or less than 10 mm in maximum diameter is called microcarcinoma, wherein no significant changes in morphology emerge. Therefore, it is necessary to further evaluate the relationship between stiffness and the pathological components in the small thyroid nodules in future study.

In this study, there were two kinds of situations leading to misdiagnosis cases of SWS parameter. On one side, the E-mean value decreased because of the signal loss like “zero signal intensity”. On the other side, many malignant nodules equal to or less than 10 mm were included, which might show low stiffness. Benign elastography features among those carcinomas may lead to false-negative SWS parameters.

There were several limitations in our study. Firstly, selection bias may exist because all patients enrolled in this study were scheduled for FNA or for surgery. That is to say, this population is not representative of a screening population, our population have a higher rate of malignancy. Secondly, the number of enrolled nodules was small and this study only reflected a single center's experience. Therefore, prospective multi-centre study with a large population from various regions and institutions, especially in those with different thyroid cancer risks, is necessary for further evaluation. Thirdly, in SWS imaging, as in other elastography techniques, the pressure applied by the probe increases the tissue's stiffness [30]. Therefore, operators need to be trained to be qualified in performing thyroid elastography before interpreting or documenting the exams. Besides, this study has not yet evaluated the influence of inflammatory diseases in thyroid. Any focal inflammatory areas should be included in the differential diagnosis of carcinoma because they are stiffer than normal thyroid tissue. Additionally, the combination of SWS imaging and conventional US should be evaluated in future study.

In summary, SWS imaging is a promising technique in the prediction of malignant thyroid nodules and E-max with SWS measurement is the strongest independent predictor for thyroid malignancy.s

## MATERIALS AND METHODS

### Patients

This retrospective study was approved by the institutional review board of the university hospital and informed consent was obtained for all the participating patients to include their data for scientific analysis. From Jan 2016 to May 2016, a total of consecutive 353 patients diagnosed with suspicious thyroid nodules underwent conventional US and SWS imaging examination before FNA or surgery. The inclusion criteria for TNs were as follows: (A) TNs were detected by conventional US or palpable by clinicians; (B) the diameter of TNs is ≥ 5 mm with enough thyroid tissue surrounding the nodule at the same depth, although some reviewers do not suggest biopsy or surgery for thyroid nodules less than 1 cm, patients who suffered from suspicious thyroid nodules were anxious despite the fact the nodules were less than 1 cm and some of them accepted FNA or surgery; (C) solid TNs or predominant solid nodules (cystic part < 25%); (D) TNs with histopathological results or follow-up period more than 6 months for those with initially benign cytological results on FNA. Finally, 31 patients were excluded for the following reasons: (A) with previous treatment such as surgery (*n* = 15); (B) US or elastography images were incomplete (*n* = 16).

Finally, the patients included 79 men and 243 women. The patient age ranged from 20.0 to 78.0 years old and the mean age was 51.2 ± 12.8 years old. For the patients with multiple nodules, the most suspicious TN was determined based on conventional US findings such as micro-calcifications, solid composition, hypoechogenicity, taller than wide shape, and poorly-defined margin or absence of halo sign [19], otherwise the largest solid one was selected. In general, only one nodule was selected for detection in each patient. Finally, 322 nodules were selected and the diameter of the nodules ranged from 5.0 mm to 56.0 mm (mean, 13.7 ± 8.7 mm).

### Conventional US

All US and SWS imaging examinations were performed with the same US machine (Aplio500, Toshiba Medical Systems Corporation, Tochigi, Japan). A 14L5 linear array transducer with frequency range of 5 to 14MHz was used for all the conventional US and SWS imaging examinations. All the patients were examined by one of three board-certified operators with sufficient experience in conventional US and US elastography, while blinded to the clinic data.

All patients underwent conventional US examination, including conventional US and color Doppler US. Firstly, all the patients were asked to lie in a supine gesture with dorsal flexion of the neck. The frequency, gain, focus position and depth were adjusted appropriately to ensure that the nodules were displayed completely and obviously on the screen. Then the target nodule and surrounding thyroid tissue were scanned transversely and longitudinally. The maximum diameter of nodule was measured on US. All the US images were stored in the internal hard disk of the US machine for subsequent analysis.

### SWS imaging

SWS imaging examination was performed thereafter by the same operator using the same transducer after conventional US. The patients were asked to hold their breath and swallowing for a few seconds when performing SWS imaging. The transducer gently touched the skin surface over the thyroid and sufficient gel was applied to form an isolation gap between the transducer and the neck so as to avoid unintentional pre-compression, which is a crucial point for SWS measurement as even a slight transducer pressure may significantly increase tissue stiffness.

**Figure 3:**
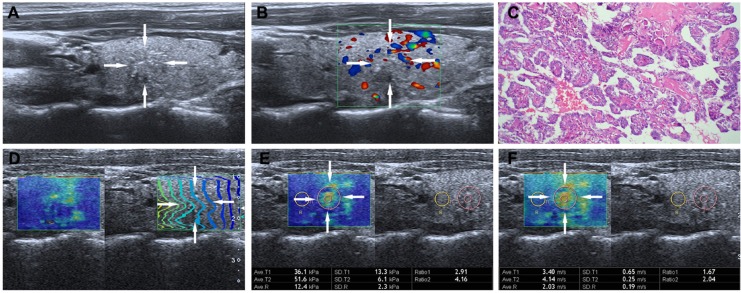
Images in a 26-year-old woman with papillary thyroid carcinoma (**A**) The nodule (arrows) is shown on conventional US, 9.6mm ×7.3mm in size in the left lobe of the thyroid appears to have iso-echoic, poorly defined margin, irregular shape, and with micro-calcifications. (**B**) The nodule (arrows) is shown on color-Doppler ultrasound, peri-nodular blood flow. (**C**) (Haematoxylin-eosin stain, original magnification, ×200) Surgery-proven papillary thyroid carcinoma. (**D**) On the right, the nodule (arrows) shows regularly parallel contour lines on the shear wave propagation mode. (**E**) The E-mean, E-max of the nodule (arrows) expressed in kPa on SWS imagine are 36.1 kPa and 51.6 kPa, respectively. (**F**) The E-mean, E-max of the nodule (arrows) expressed in m/s on SWS imagine are 3.40 m/s and 4.14m/s respectively.

**Figure 4:**
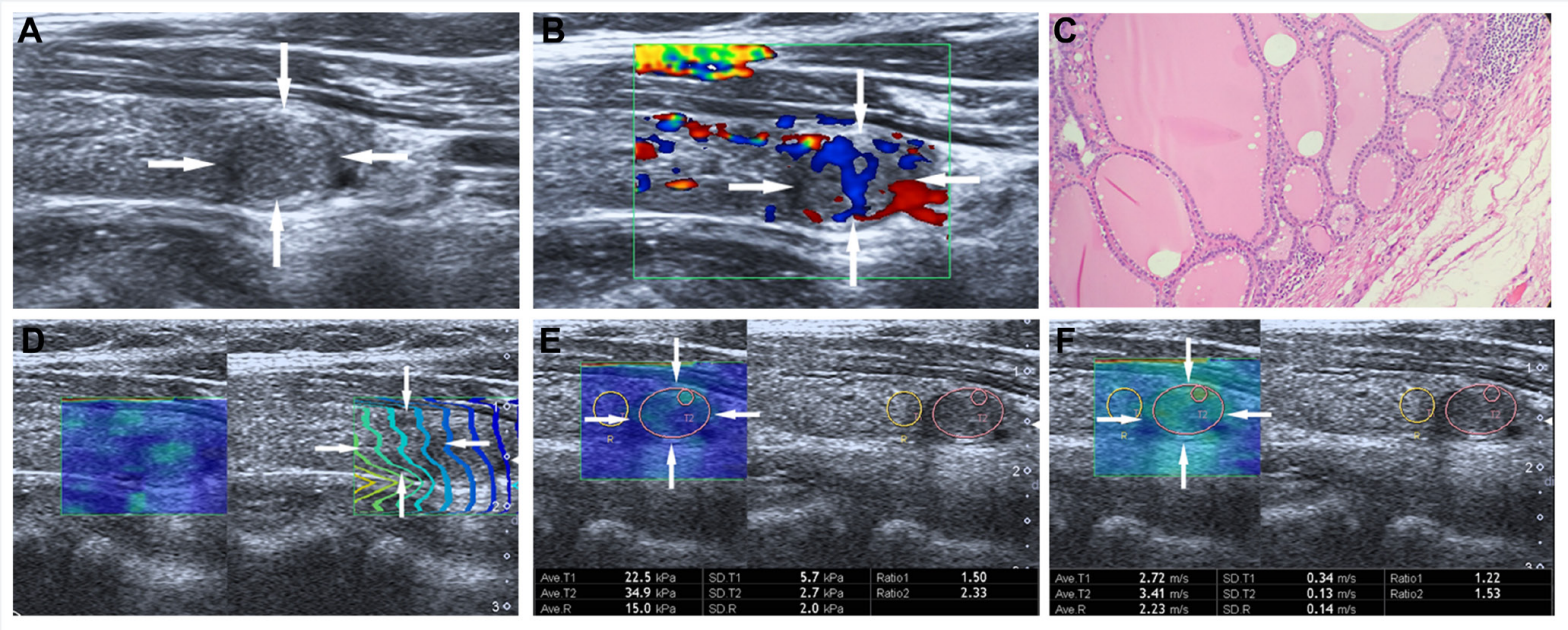
Images in a 55-year-old woman with nodular goiter (**A**) The nodule (arrows) is shown on conventional US, 10.1 mm × 6.8mm in size in the left lobe of the thyroid appears to have hypoechogenicity, well defined margin, regular shape, and without calcification. (**B**) The nodule (arrows) is shown on color-Doppler ultrasound, intra-nodular and slight peri-nodular blood flow. (**C**) (Haematoxylin-eosin stain, original magnification, ×200), Surgery-proven nodular thyroid goitre. (**D**) On the right, the nodule (arrows) shows regularly parallel contour lines on the shear wave propagation mode. (**E**) The E-mean, E-max of the nodule (arrows) expressed in kPa on SWS imagine are 22.5kPa and 34.9 kPa, respectively. (**F**) The E-mean, E-max of the nodule (arrows) expressed in m/s on SWS imagine are 2.72 m/s and 3.41 m/s respectively.

The reliability of the data is guaranteed by observing the contour lines within the ROI in the propagation mode. When the contour lines are parallel, the shear waves propagate properly and the reliability of the obtained data is high. Conversely, the contour lines are distorted and not parallel to one another, the reliability of the obtained data is low. The intervals between the displayed contour lines are wider in stiff tissues and narrower in soft tissues. The sampling box of the desired size is set in the color map, which is moved to include the target lesion and some surrounding thyroid tissue. Then the speed mode is initialized and the shear wave propagation speed is measured quantitatively, in which blue and red areas correspond to softer and stiffer regions, respectively. One 2-mm sized circular region of interest (ROI) was located over the stiffest area in the lesion and one large ROI over the whole lesion. Another 2-mm sized ROI was placed on the surround thyroid tissue at the same depth. The interval between two performances was about 5 s, and the operator chose the optimal images to freeze and store in the machine. Subsequently, the operator switched to elasticity mode to gain the results of elasticity modulus measurement. The criteria for the 2-mm ROI selection in the TN was as follows: (i) ROI was placed on the solid portion of the nodule, especially in the cystic-solid nodules; (ii) calcified and liquefaction component of the nodule was avoided; (iii) the adjacent thyroid tissue was not included in ROI. Usually, the whole operation process was repeated three times and only the optimal images were saved. E-mean and E-max values of SWS imaging for the longitudinal plane were evaluated: (1)E-mean, mean value with elastic modulus (kPa) or SWS (m/s) in the largest ROI; (2) E-max, maximum value (kPa or m/s) in the stiffest area in the 2-mm ROI. The T-SWS imaging speed and elastic modulus values ranged from 0–8 m/s and 0 – 180 kPa respectively (Figure 3 and 4).

### Image interpretation

Another two board-certified readers retrospectively analyzed the images, who were blind and without access to relevant clinical information and final diagnoses. Disagreement was solved by consensus. Both readers had been trained to review the images before the study. Under the auspices of the American College Radiology(ACR), a practical and standard lexicon for describing the US characteristics of thyroid nodules was developed. The US features included nodule echogenicity, shape, margin, composition and calcification. The echogenicity was classified as marked hypoechogenicity (decreased echogenicity relative to adjacent neck musculature), hypoechogenicity (less echogenicity compared with surrounding thyroid tissue), isoechogenicity (echogenicity equal to surrounding thyroid tissue) and hyperechogenicity (more echogenicity compared with surrounding thyroid tissue). The shape was categorized into taller-than-wide (a ratio of >1 in the anteroposterior diameter to the horizontal diameter when measured in the transverse plane) or wider-than-tall. The margin was classified into poorly-defined, smooth, irregular, lobulated or extrathyroidal extension. The internal composition of nodule was classified into solid, predominately solid, predominately cystic and spongiform. Micro-calcification was defined if calcification was equal to or less than 1mm in diameter or detected tiny, spot fo ci with or without acoustic shadows. Macro-calcification was defined if calcifications become large enough to result in posterior acoustic shadowing. Peripheral calcifications was defined if calcifications occupy the periphery of the nodule. If both micro-calcification and macro-calcification were present in the same nodule, it was referred as micro-calcification [31]. The color Doppler US patterns of the lesions were classified into three types: type I, absence of color signal; type II, peri-nodular and absent or slight intra-nodular blood flow; or type III, marked intra-nodular and absent or slight peri-nodular blood flow [32].

### Inter-operator and intra-operator consistency of SWS imaging

To assess inter-operator consistency, another 30 consecutive patients with 30 thyroid nodules were enrolled. All performances were conducted by another two independent operators who had similar experience on SWS imaging and were blinded to each other's measurements while performing SWS imaging in the same day. To evaluate the intra-operator consistency, it was tested by the same operator and repeated the same performance with one day interval. All performances were conducted in line with the method described above and the cases were excluded in the final diagnostic efficiency analysis.

### Reference standard

The reference standard was diagnostic FNA cytology or histology. Histopathologic data were obtained from the medical records of subjects after thyroidectomy with the reporting pathologist blinded to the results of conventional US and elastography examinations. All FNA reports were reviewed by a cytopathologist who specialized in thyroid cytopathology for 3 years. The diagnostic cytology reported was following with the Bethesda system for reporting thyroid cytopathologic findings and the duration of imaging follow-up with US for the nodules with initially benign FNA results was at least 6 months (range: 6–24 months), which was based on the recommendation from ATA guideline [19, 33, 34].

### Statistical analysis

Statistical analysis was performed by using SPSS, version 22.0 (SPSS, Chicago, IL). Receiver operating characteristic (ROC) curve analysis was performed by using MedCalc for Windows (version 12.2.0.0; MedCalc Software, Mariakerke, Belgium). Descriptive statistics were applied to all collected variables expressed as frequency tables for categorical data or mean values ± standard deviations for continuous data. Student's *t*-tests or Mann–Whitney tests were used to assess the differences between two groups of quantitative variables. ROC curve was applied to obtain AUC. Sensitivity, specificity, PPV, NPV and accuracy were calculated for the diagnosis of malignant nodules with conventional US and SWS imaging in all nodules, as well as in nodules categorized into different sizes. The optimal cut-off values were obtained by using the Youden index (maximum of sensitivity + specificity). For the comparisons of sensitivity and specificity, the McNemar test was used. Link between these two qualitative parameters was estimated using a chi-square test or Fisher's exact test. Comparisons of AUC among four SWS imaging indices (*i.e*., E-max in kPa or m/s, E-mean in kPa or m/s) were conducted by the method of univariate *Z* score test to evaluate the diagnostic performance of each elasticity parameters in all and subgroup nodules with different sizes [35]. For the correlations between SWS imaging parameters and nodule sizes, Spearman's correlation coefficient analysis was applied. Logistic regression analysis was used to assess independent factors for predicting malignancy of each suspicious conventional US finding and each optimal cut-off value on SWS imaging. For all analysis, the tests were two-sided and a significance level of α = 5 %, *P-value* < 0.05 was considered statistically significant. Reader agreement among the 2 operators in classifying conventional US and SWS imaging was estimated using the kappa analysis to assess the intra-operator and inter-operator reproducibility of SWS imaging performances. Agreement was graded as poor (k < 0.20), moderate (k = 0.20 – 0.40), fair (k = 0.40 – 0.60), good (k = 0.60 – 0.80), or very good (k = 0.80 – 1.00) [36]. Finally, to investigate whether the nodule size would affect the diagnostic performance of SWS imaging, the nodules were divided into three groups according to nodule size: group 1, ≤ 10 mm; group 2, 11 – 20 mm; group 3, > 20 mm. ROC curve analysis was performed to evaluate the diagnostic performance (*i.e*., area under the ROC curve, A_Z_) regarding the three subgroups datasets in distinguishing benign thyroid nodules from malignant ones.
